# Insight Is Not in the Problem: Investigating Insight in Problem Solving across Task Types

**DOI:** 10.3389/fpsyg.2016.01424

**Published:** 2016-09-26

**Authors:** Margaret E. Webb, Daniel R. Little, Simon J. Cropper

**Affiliations:** Melbourne School of Psychological Sciences, The University of MelbourneMelbourne, VIC, Australia

**Keywords:** insight, problem solving, accuracy

## Abstract

The feeling of insight in problem solving is typically associated with the sudden realization of a solution that appears obviously correct (Kounios et al., 2006). Salvi et al. (2016) found that a solution accompanied with sudden insight is more likely to be correct than a problem solved through conscious and incremental steps. However, Metcalfe (1986) indicated that participants would often present an inelegant but plausible (wrong) answer as correct with a high feeling of warmth (a subjective measure of closeness to solution). This discrepancy may be due to the use of different tasks or due to different methods in the measurement of insight (i.e., using a binary vs. continuous scale). In three experiments, we investigated both findings, using many different problem tasks (e.g., Compound Remote Associates, so-called classic insight problems, and non-insight problems). Participants rated insight-related affect (feelings of Aha-experience, confidence, surprise, impasse, and pleasure) on continuous scales. As expected we found that, for problems designed to elicit insight, correct solutions elicited higher proportions of reported insight in the solution compared to non-insight solutions; further, correct solutions elicited stronger feelings of insight compared to incorrect solutions.

## Introduction

*Insight* or *Aha* is often identified as the subjectively distinct feeling of sudden and unexpected understanding that may accompany attempts to solve a problem (Sternberg and Davidson, 1995; Davidson and Sternberg, 2003; Cushen and Wiley, 2012; Weisberg, 2014). This feeling of sudden comprehension often alerts the problem solver to a potentially correct solution (Irvine, 2015). However, as noted as early as Poincaré (1913), feelings of *Aha* may also accompany ideas that turn out to be incorrect. Recent investigations into the relationship between Aha and accuracy indicate that the Aha experience predicts accuracy (Salvi et al., 2012, 2016); however, these investigations focus on non-classic insight problems (i.e., problems such as Compound Remote Associates, Rebus Puzzles, and Anagrams, as opposed to the classic riddle-like problems favored by Gestalt psychologists that typically populate the insight problem solving literature). In this paper, we compare newer “non-classic” insight problems with the classic problems and non-insight problems and explore the accuracy-Aha relationship across problem types.

Definitions of *insight* vary on three dimensions: process, task, and phenomenology (Öllinger and Knoblich, 2009). *Process* concerns the cognitive mechanisms through which insightful solutions are generated. Descriptions of an *insightful* problem solving process emphasize a sudden certainty of a correct response, with little or no conscious access to the processing of the solution, whereas an *analytic* process emphasizes a deliberate and systematic evaluation of the problem, emphasizing logical deduction and strategic thinking (Kounios et al., 2008; Topolinski and Reber, 2010).

The *task* dimension concerns the identification of tasks that elicit sudden (insight) solutions; these tasks are often identified through comparison to other tasks that require stepwise solutions (Öllinger and Knoblich, 2009). The concept of a *problem space* iin which all possible problem states are mapped provides a useful tool for differentiating problem types. A regular problem has a *well-defined* problem space with operators that are obvious enough to enable steady stepwise progress toward the solution (DeYoung et al., 2008). In contrast, an *ill-defined* problem does not allow a clear mapping of the initial problem space, and the method of achieving the solution is unclear. Indeed, an ill-defined problem often deliberately extends the problem space by misdirecting the solver (Ovington, 2016). For these problems, insight is often described as a restructuring of ill-defined problem space, which occurs after a period of impasse. The sudden narrowing of the problem space enables an easier generation of a solution. Classic insight tasks are typical of ill-defined problems (DeYoung et al., 2008).

The *phenomenology* of insight focuses on the Aha experience and is typically examined using case studies and anecdotes; however, there has been a recent push to explore the phenomenology using self report in laboratory studies (e.g., Bowden et al., 2005; Danek et al., 2014b). The use of the term *insight* is inclusive of the *Aha experience* as well as other insight related affect, such as confidence, impasse, surprise and pleasure, proposed to accompany an Aha experience (Danek et al., 2014a).

### Insight problems and problems thereof

The methodological challenges of objectively measuring a subjective phenomenon such as insight are well known (Öllinger and Knoblich, 2009; Ash et al., 2009). Historically, researchers have used “classic insight problems,” ill-defined problems originally used by Gestalt psychologists to elicit feelings of insight upon realization of the solution. Gestalt psychologists investigated insight as the result of perceptual and cognitive *restructuring* (Klein and Jarosz, 2011). This is of note predominately because the Gestaltists had backgrounds in visual science and were particularly invested in perceptual restructuring, which is a sudden change in which an object is perceived (say in Rubin's Face/Vase illusion, where the perception shifts from figure/face to ground/vase). Similarly, much insight research revolves around *cognitive restructuring*, a sudden change in the way a problem is perceived. Restructuring the problem makes the correct solution easy to obtain. This sudden ease of solution results in the feelings of pleasure, joy, and the rise of confidence associated with insight.

An example of a classic insight problem follows:

Water lilies double in area every 24 h. At the beginning of summer there is one water lily on the lake. It takes 60 days for the lake to become completely covered with water lilies. On which day is the lake half covered? (Sternberg and Davidson, 1982).

The answer (59) may or may not be immediately apparent: problem solvers frequently fixate on what they perceive as the key information of “60 days,” and “half full” and either begin calculating the answer from day 1 or conclude that the answer is 30 (Bowden, 1997). For others, it becomes immediately apparent that, if water lilies double in area every 24 h and that if the lake is full on day 60, it must be half full on day 59 (see Appendix for other example problems). This question functions as an insight problem only if the solver initially misconstrues the problem space (focusing on the information of “60” and “half-full”). Sudden realization of the solution is accompanied by a feeling of certainty, as the answer is simple to check. For a comprehensive review of this style of research, see Sternberg and Davidson (1995).

*Non-insight problems* have been used as a control for insight problems, particularly when contrasting individual differences in problem solving (e.g., Fleck, 2008; Gilhooly and Fioratou, 2009; DeCaro et al., 2015). Non-insight problems are designed to be solvable through a process of systematic application of knowledge and logical deduction (Bowden, 1997, p. 548). For example:

If you have four coins, two slightly heavy and two slightly light, but which look and feel identical, how could you find out which are which in two weighings on a balance scale? (Schooler et al., 1993).

The answer to this question requires systematic consideration of the problem space, and potential steps toward solving the problem. The solution: (1) place one coin either side (if they do not balance, you have identified one heavy and one light coin), (2) replace one coin with one of the remaining. This weighing will provide the remaining information.

This problem and problems like it tend to be categorized as a non-insight problems (Gilhooly and Murphy, 2005). However, for someone with little or no training in logic, the underlying mechanisms may involve the experience of insight. The Aha experience may arise from recognizing that one cannot complete this task in two weighings if one attempts to weigh all four coins at once. From a from a phenomenological perspective, for experienced puzzle solvers, it is possible that neither or possibly both the heavy/light coin problem and the problem of the lilies raised above may be considered insight problems (Bowden, 1997). In other words, classic insight and non-insight problems alike can be solved with both insight *and* analysis (Weisberg, 2014). In the absence of feedback from the problem solver or other kinds of compelling evidence, the previously held *a priori* assumption that particular problems elicit insight, and are solved using particular processes (i.e., insightful or analytic), is highly problematic. Though classification and use of non-insight problems stems largely from the seminal papers of Metcalfe and Wiebe (1987) and Weisberg (1995), these authors noted the vagueness of the distinction between insight and non-insight problems, and there has been little systematic investigation of insight and non-insight problem types since the publication of those papers.

While research historically has contrasted classic insight problems with non-insight problems, more contemporary research uses a single problem task as indicative of both insightful and non-insightful problem solving, relying on the participant's self-report to determine whether or not insight has occurred (e.g., Bowden, 1997; Bowden and Jung-Beeman, 2003a; MacGregor and Cunningham, 2008). Non-classic problems (such as Compound Remote Associates; Bowden and Jung-Beeman, 2003b), rebus puzzles (MacGregor and Cunningham, 2008), or anagrams (Novick and Sherman, 2003; Salvi et al., 2016), are presented to participants, who provide a solution and also information about their experience of insight. This shift to asking whether or not insight was experienced in these non-classical problems was sparked by Bowden and Jung-Beeman (2003a) and Jung-Beeman et al. (2004), and has been followed by a line of research largely centered around Compound Remote Associates (CRAs; Bowden and Jung-Beeman, 2003b).

The taxonomy of these problem tasks as *pure insight, non-insight* or as both insight and non-insight (*hybrid*) has been debated for decades (see particularly Metcalfe and Wiebe, 1987; Weisberg, 1995 for contrasting viewpoints). Yet there is little recent evidence regarding the efficacy of classic (or proposed *pure*) problems to elicit insight or, lack thereof, consistently. There is also little investigation regarding the effect of accuracy on insight.

### Accuracy and Aha

Salvi et al. (2016) investigated four types of non-classic insight problems (as classified by Cunningham et al., 2009): CRAs, Rebus Puzzles, Anagrams, and Visual Puzzles and found that insightful processes elicited a higher proportion of correct responses. The authors had solvers use a dichotomous indication of whether or not the problem had been solved insightfully or analytically. Danek et al. (2016) used a similar dichotomous measure, investigating three widely used classic insight problems, and found that, across all problem types, participants reported experiencing insight in only 51.9% of correctly solved trials. Two questions arise from these studies: (1) are there differences between classic and non-classic problem types in the degree of insight elicited in the solution of each task, (2) are there differences between so-called insight and non-insight problems, and (3) what influence does solution accuracy have on the experience of insight?

Methods of insight self-report have varied between: dichotomous (insight/analytic) responses (Danek et al., 2016; Salvi et al., 2016), Likert scales (Bowden and Jung-Beeman, 2003a), and rating scales (0–100; Danek et al., 2014a), but have typically been analyzed as reports of insight (or insightful processing) vs. non-insight (or analytical processing). The strength of an insight response and its relationship with other significant components of insight phenomenology (such as a feeling of Impasse, and Confidence) are yet to be examined in depth (Danek et al., 2014a, 2016).

Given the differences in the purported solution methods, ill-defined problems such as classic insight problems and CRAs may elicit greater amounts of insight compared to well-defined problems such as non-insight problems. Nonetheless, non-insight problems may also evoke insight in their solution, though this may only be evident when insight is measured on a continuous scale. Given the clarity of the problem space in non-insight problems, performance accuracy may be higher when problem solving is time-constrained in non-insight problems compared to insight problems or CRAs.

### The present research

The current study investigated ratings of insight (the Aha experience and other insight-related affect: i.e., confidence, surprise, impasse, and pleasure; Danek et al., 2014a) and performance accuracy in order to examine the relationship between accuracy and Aha across problem types (insight problems, non-insight problems, and CRAs). We were interested in the differences in performance accuracy and Aha ratings across problem types, and predicted that there would be (1) higher accuracy rates on non-insight problems compared to insight problems and CRAs, and (2) there would be significantly higher ratings of Aha in insight problems and CRAs compared to non-insight problems. We predicted that feelings of Aha would be predictive of correct solutions in classic insight problems and CRAs but that ratings of Aha would not be predictive of correct solutions in non-insight problems.

## Experiment 1

### Methods

#### Participants

University of Melbourne students (193: 118 female, age range, 17–52, mean, 19.639) completed the study for course credit. Before beginning the study, participants were provided with consent forms detailing the proposed study. Nine participants were removed for errors on more than 20% of the tasks.

#### Materials

##### Problem solving tasks

Insight in problem solving was measured with a mixture of “classic” insight and non-insight problems, and compound remote associates (CRAs; Bowden and Jung-Beeman, 2003b).

##### “Classic” insight and non-insight problems

Riddle tasks and brain teasers were drawn from the existing insight problem solving literature (e.g., Schooler et al., 1993; Gilhooly and Murphy, 2005; Karimi et al., 2007), and categorised as insight and non-insight problems based on their classification in previous studies (see Appendix 1 for problems). Participants were given 4 min per problem to generate solutions. Accuracy and RT were recorded.

##### Compound remote associates

CRAs (Bowden and Jung-Beeman, 2003b) are verbal association tasks patterned after the Remote Associates Test (RAT: Mednick, 1962). Three words are presented to the participant, each of which can be combined with a fourth word to make compound words (e.g., *potato/tooth/heart* can all be combined with *sweet*). CRAs were developed in response to criticisms of classic insight problems, particularly the limited number of problems and the need for different types of problems (incrementally solvable, “non-insight problems”) used as a control. Participants had 30 s to generate the fourth word.

#### Procedure

Each participant was individually tested: problems sets and questionnaires were presented in random order. No solutions were given.

##### Problem solving sets

There were two problem-solving sets: classic and non-classic problem solving, respectively. The classic “insight” and “incremental” (non-insight) problems were randomly interleaved within a set. Participants were given no information about whether the problem to be solved was classified as “insight” or “non-insight” but were given 4 min to work through the problem. In the CRA problem set, 20 problems, selected for varying difficulty levels (Bowden et al., 2005) were presented in random order. Five practice trials preceded the set. Participants were given 30 s to solve the word association task.

Before the problem solving set, participants were given the following information (drawn from Danek et al. (2014b):

*We would also like to know whether you experienced a feeling of insight when you solve each task: A feeling of insight is a kind of “Aha!” characterized by suddenness and obviousness (and often relief!)—like a revelation. You are relatively confident that your solution is correct without having to check it. In contrast, you experienced no Aha! if the solution occurs to you slowly and stepwise. As an example, imagine a light bulb that is switched on all at once in contrast to slowly dimming it up. We ask for your subjective rating whether it felt like an Aha! experience or not, there is no right or wrong answer. Just follow your intuition*.

After each problem solving task, participants rated five feelings during the problem solving task: (1) Confidence that the given response was correct (“very unsure” to “very sure”), (2) *Strength* of the insight experience (“very weak” to “very strong”), (3) *Pleasantness* of the insight experience (“very unpleasant” to “very pleasant”), (4) *Surprising* nature of the insight experience (“not surprising” at all to “very surprising”), (5) Feeling of *impasse* before the insight experience (“no impasse” at all to “very stuck”)^1^. Participants responded by moving a slider (pre-set at 50) along a scale of 0–100.

##### Questionnaires

A series of individual differences measures were presented in random order. These included the *O-LIFE* (Oxford-Liverpool Inventory of Feelings and Experiences; Mason and Claridge, 2006), Raven's (1985) *Advanced Progressive Matrices*, a verbal fluency measure adapted from Lezak (2004), and an adaptation of the Alternative Uses Task (AUT: Guildford et al., 1978). These measures are reported elsewhere in a follow-up study of the same sample.

## Results and discussion

### Descriptive statistics

Problems were scored as either correct or incorrect and averaged across category (insight, non-insight, CRAs), as were the ratings of insight related affect. Descriptive statistics of performance accuracy, and ratings of insight-related affect are displayed in Table 1.

**Table 1:** **Means and standard deviations for accuracy and insight quale for classic insight and non-insight problems, and for compound remote associates (CRAs)**.

	**Experiment 1**		**Experiment 1a**		**Experiment 2**	
	***M***	***SD***	***M***	***SD***	***M***	***SD***
**CLASSIC INSIGHT PROBLEMS**						
Accuracy	0.51	0.28	0.49	0.25	0.45	0.30
Aha	45.16	21.05	49.25	25.25	42.47	18.11
Confidence	56.61	23.10	55.01	23.44	52.30	22.57
Impasse	54.04	21.02	57.63	15.99	55.27	17.40
Surprise	34.63	19.37	41.52	14.68	41.50	13.45
Pleasure	50.69	23.20	53.64	18.78	51.54	14.95
**NON-INSIGHT PROBLEMS**						
Accuracy	0.54	0.25	0.64	0.19	0.56	0.27
Aha	35.00	16.40	64.40	19.25	36.34	18.11
Confidence	59.56	21.58	57.47	19.37	48.15	18.36
Impasse	50.49	18.96	48.52	16.04	49.85	18.55
Surprise	30.35	16.22	36.18	18.69	37.25	15.64
Pleasure	49.63	17.63	50.70	15.57	51.07	14.49
**CRAs**						
Accuracy	0.37	0.20	0.45	0.20	0.47	0.16
Aha	30.66	13.81	41.50	20.34	38.45	13.11
Confidence	40.93	15.79	45.33	17.76	44.74	14.07
Impasse	59.92	14.19	63.01	15.87	55.35	15.04
Surprise	28.31	14.77	42.56	18.21	38.66	11.86
Pleasure	36.17	18.23	49.64	13.50	48.31	11.84

We include in our results the Bayes Factor (*BF*_10_), which compares the ratio of model evidence for the alternative hypothesis (i.e., that there is an effect) to the null hypothesis^2^. This enables us to provide a more nuanced picture of the data in relation to the question addressed by the experiment than a standard *p*-value (Wagenmakers et al., 2016).

#### Accuracy and insight: differences across problem types?

**Figure 1:**
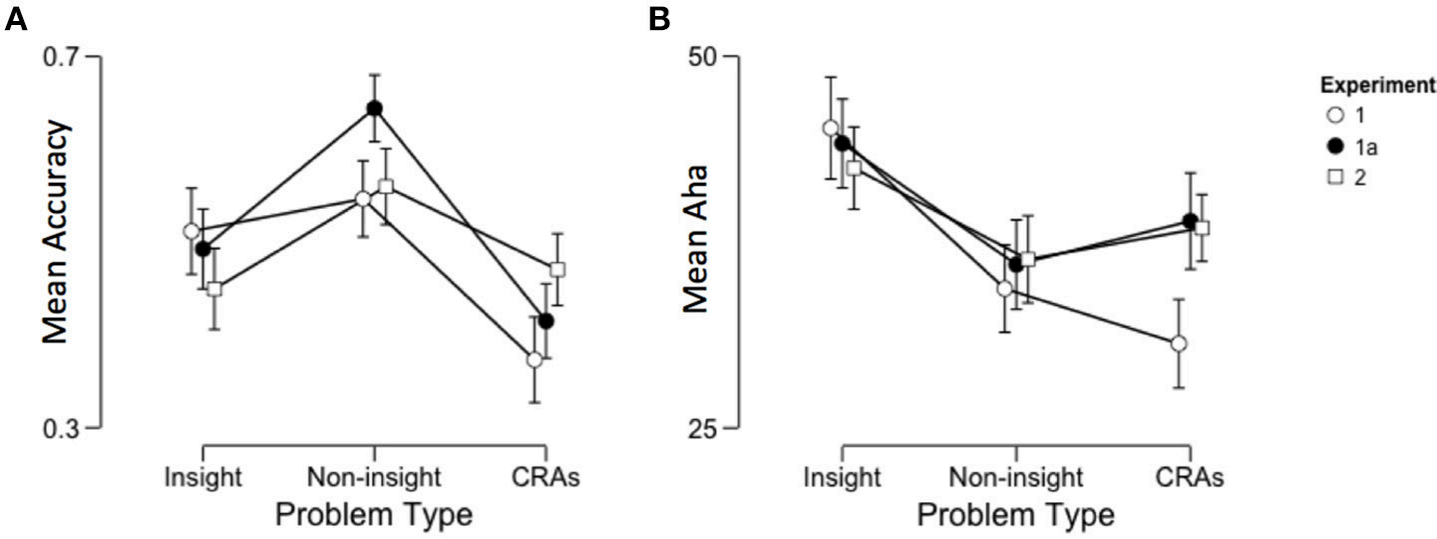
**Mean (A) accuracy and (B) reported insight across problem types, with separate lines representing the different experiments (Experiments 1a and 2 have participants are filtered for ESL students)**. Error bars are 95% confidence intervals.

The first question was whether the accuracy of insight and non-insight problem solving differed across problem types (classic-insight, classic-non-insight and non-classic insight). A Bayesian repeated-measures ANOVA indicated strong evidence for a difference in accuracy of response between problem types, *BF*_10_ >150, η^2^ = 0.22. The effect was largely explained by low accuracy on CRAs compared to insight (mean difference = 0.14, *BF*_10_ > 150) and non-insight (mean difference = 0.17, *BF*_10_ > 150) problems (see Figure 1A). There was no difference between insight and non-insight problems (mean difference = 0.035, *SE* = 0.032, *p* = 0.81). The low accuracy on CRAs is congruent with Bowden and Jung-Beeman's (2003b) normative data, from which the problems were drawn.

A second Bayesian repeated-measures ANOVA (see Figure 1B) indicated strong evidence for difference between problem types on the level of reported insight, *BF*_10_ > 150. *Post-hoc* analyses indicated that the effect was driven by higher reported insight in insight problems compared to both non-insight problems (mean difference = 10.15, *BF*_10_ > 150) and CRAs (mean difference = 14.49, *BF*_10_ > 150). There was no significant difference between CRAs and non-insight problems (mean difference = 4.34, *BF* = 1.562). The difference between insight and non-insight problems is congruent with the literature indicating that these are solved with different underlying processes (Gilhooly and Murphy, 2005; Chu and MacGregor, 2011). The difference in ratings of insight between insight problems and CRAs speaks against the use of CRAs as insight problems; however, this may simply indicate that CRAs are a hybrid insight problem (i.e., CRAs may be used as both insight and non-insight problems, depending on self-reported classification, e.g., Bowden and Jung-Beeman, 2003a; Salvi et al., 2012). It may also reflect the reduced solution accuracy.

## Accuracy and insight affect: relationships

**Figure 2:**
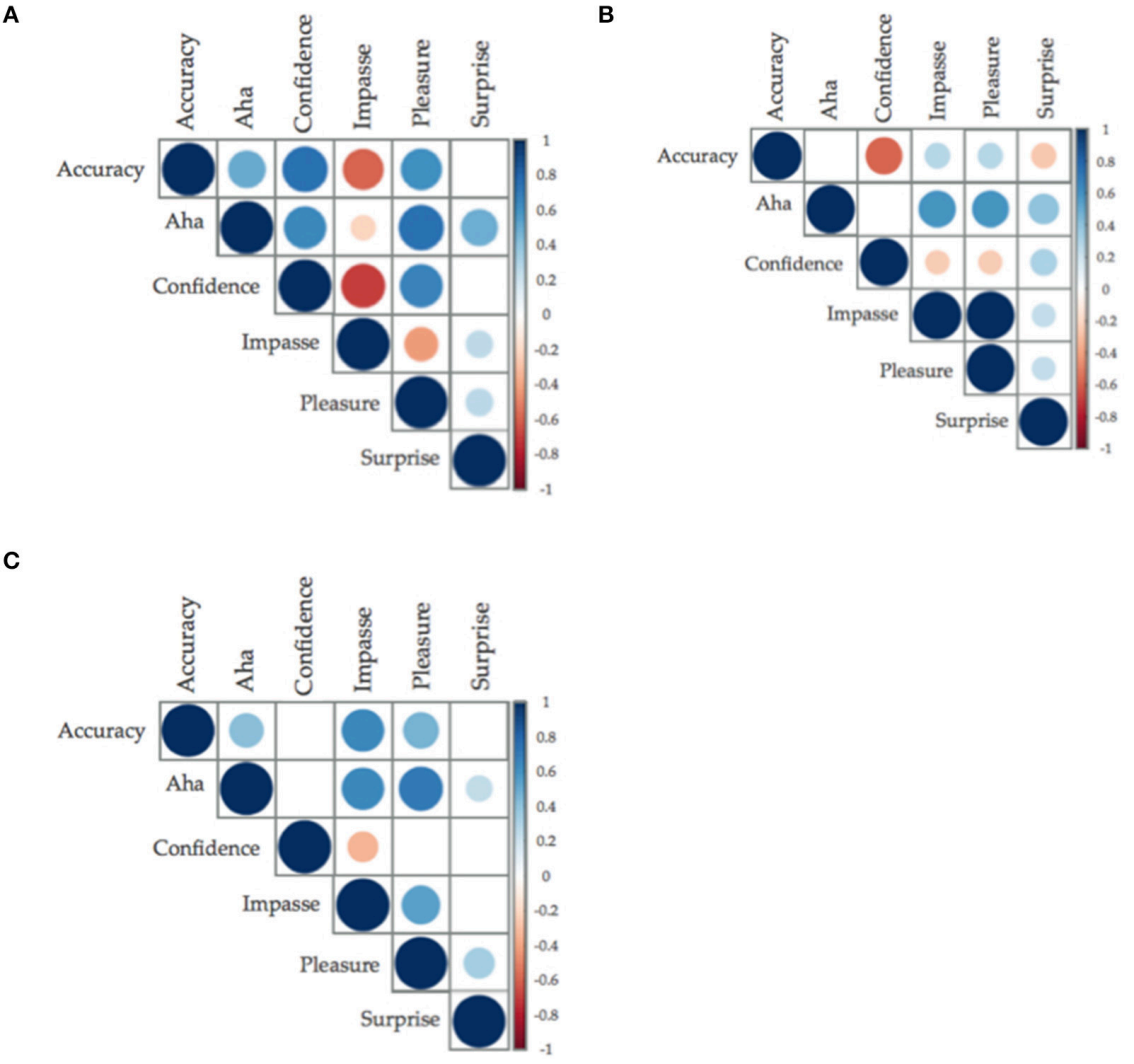
**(A–C)** Correlation plots between accuracy and insight and insight related affect. Size of the circle and saturation of color determine the strength of the correlation; the color determined the direction of the relationship, with positive being blue (**A**: classic insight problems; **B**: classic non-insight problems; **C**: compound remote associates. Only relationships with less than *p* = 0.05 have been graphed).

Pearson correlations suggest that accuracy is related to degree of reported Aha for insight problems and CRAs but not for non-insight problems (see Figure 2). There were moderately strong, significant and positive relationships between feelings of Aha and accuracy on both classic insight problems (*r* = 0.50, *BF*_10_ > 150) and CRAs (*r* = 0.41, *BF*_10_ > 150); however, there was no relationship between accuracy and non-insight problems (*r* = 0.02, *BF*_10_ < 1). This relationship supports the current assumptions within insight problem-solving literature that insight problems result in feelings of insight in their accurate solution but that non-insight problems do not (Gilhooly and Murphy, 2005; Chu and MacGregor, 2011). The relationship between accuracy and Aha on CRAs suggests that the difference between insight problems and CRAs was indicative of a lack of accuracy, rather than the use of CRAs as hybrid problems.

Across problem types, Aha was significantly and positively related to Confidence, Pleasure, and Surprise (see Figure 2, see Supplementary Materials for correlation matrices). Confidence was the most strongly related with Aha ratings across problem types, having a moderate to strong positive relationship with Aha in insight and non-insight problems, and in CRAs. The relationship between Aha and Impasse was negative and significant for insight problems, and negative but non-significant for both non-insight problems and CRAs. Interestingly, ratings of Surprise were significantly and positively related with ratings of Aha, but not with solution accuracy. This suggests the importance of surprise in an Aha experience.

### Summary

The results of Experiment 1 support the assumptions in the literature: Aha occurred more often in insight than non-insight problems. The moderate positive relationship between Aha and performance accuracy on both classic insight problems and CRAs indicated that performance accuracy was an important component of insight affect in problem solving. This may be indicative of the sudden ease of solution once the problem space has been restructured and the solution is easy to realize.

The positive relationship between Surprise and Aha indicated that Surprise may be an important component of the Aha experience, more than the previously considered Impasse.

Low levels of accuracy potentially indicate that students with English as a second language (ESL) may have experienced more difficulty on some of the problems, as these problems require high levels of English proficiency (Ansburg, 2000).

## Experiment 1a

We sought in Experiment 1a to replicate our Experiment 1 results using a sample that was explicitly selected with English as a first language.

### Methods

#### Participants

Undergraduates from the University of Melbourne (82: 64 female, age range, 16–47, mean, 19.60) completed the study for course credit. Eight participants were removed for errors on more than 20% of the tasks.

#### Materials, procedure, and design

The materials and procedure were identical to Experiment 1, save that participants were tested online, and ESL students were requested not to participate in the study.

### Results and discussion

The results of Experiment 1a replicated the very strong support of differences between problem type on accuracy found in Experiment 1, *F*_(2, 158)_ = 33.98, *BF*_10_ > 150, η^2^ = 0.30, with *post-hoc* analyses indicating significant differences between all variables: Non-insight problems demonstrated significantly higher accuracy than both insight problems (mean difference = 0.15, *BF*_10_ > 150) and CRAs (mean difference = 0.23, *BF*_10_ > 150), and insight problems demonstrated higher accuracy than CRAs (mean difference = 0.08, *BF*_10_ = 2.387). This marks a change from Experiment 1, in which the low accuracy on CRAs alone drove the observed difference. This change in results may be arising from the filtering of ESL students.

The ANOVA conducted on the elicited Aha across problem type demonstrated strong support for differences between problem type, *F*_(2, 158)_ = 33.98, *BF*_10_ = 29.90, η^2^ = 0.084, with insight problems eliciting significantly higher ratings of insight than non-insight problems (mean difference = 6.130, *BF*_10_ = 75.662). As in Experiment 1, CRAs and non-insight problems demonstrated an anecdotal difference in reported insight (mean difference = 2.105, *BF*_10_ = 1.592); however, in another marked difference from Experiment 1, there was no difference between insight problems and CRAs (mean difference = 5.025, *BF*_10_ = 0.279). This fluctuation in results may be indicative of the “hybrid” nature of CRAs (i.e., as both an insight and non-insight problem), an indication of the filter of ESL students, or an indication of greater accuracy eliciting greater insight.

#### Investigating the relationship between accuracy and Aha

**Figure 3:**
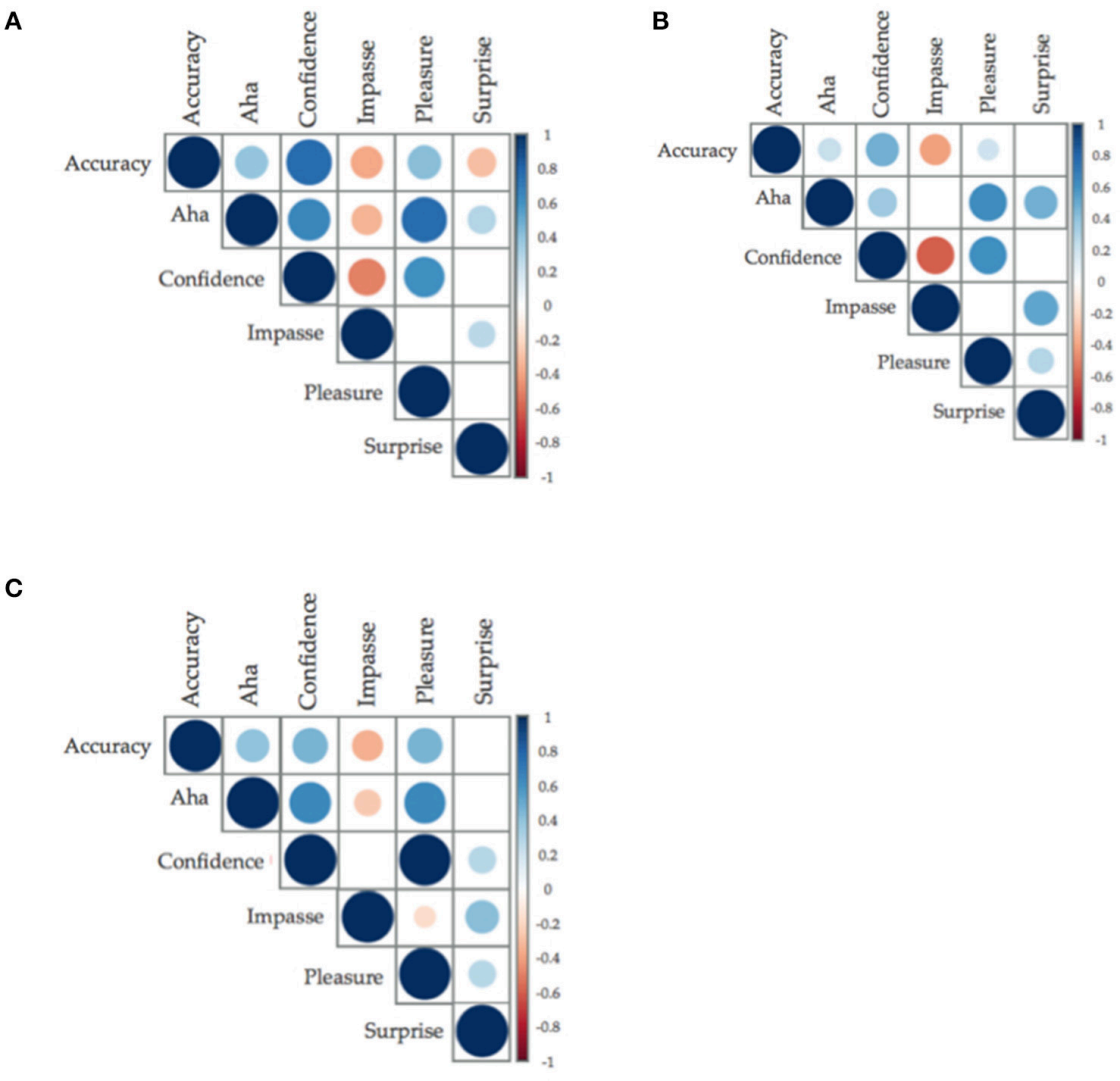
**(A–C)** Correlation plots between accuracy and insight and insight related affect. Size of the circle and saturation of color determine the strength of the correlation; the color determined the direction of the relationship, with positive being blue (**A**: classic insight problems; **B**: classic non-insight problems; **C**: compound remote associates. Only relationships with less than *p* = 0.05 have been graphed).

The moderate positive relationship between accuracy and insight affect were replicated in insight problems (*r* = 0.40, *BF*_10_ > 150) and CRAs (*r* = 0.40, *BF*_10_ = 85.65), see Figure 3; however, in non-insight problems, the relationship between accuracy and insight shifted from no relationship to a positive relationship, albeit a weak one (*r* = 0.24, *p* = 0.04, *BF*_10_ = 48.502). This marks a change from the current literature, in which non-insight problems are used as controls. However, it is congruent with statements from Weisberg (2014) indicating that both insight and non-insight problems can be solved through insightful or analytic processes.

#### Investigating relationships within insight affect

The direction of the relationships between accuracy and insight related affect were replicated, as were the direction of the relationships within insight related affect (i.e., Aha, Confidence, Impasse, Pleasure, and Surprise). The relationship of Surprise with performance accuracy and Aha ratings were again interesting in this dataset: There was a negative relationship between Surprise and performance accuracy, yet a positive weak-to-moderate with Aha. Surprise may be the component of insight related affect that is able to differentiate an Aha experience from the pleasure and confidence of a solution.

### Summary

Investigation of differences in Experiment 1a replicate the findings in Experiment 1; that is, there is a significant difference in performance accuracy and reported insight across problem types. However, the relationship between accuracy and Aha ratings reflects the growing indication that problems can be solved with and without feelings of insight.

## Experiment 2

In this study, we investigated the consistency of the relationship between reported insight and problem type by replicating the results from Experiments 1 and 1a. We also investigated the effect of feedback on reported insight; this data is not presented here as we focus instead on performance accuracy on reported insight. We replicated the analyses of Experiment 1a, and extended these analyses by combining the datasets of Experiments 1, 1a, and 2 to run a Multilevel Logistic Regression.

### Methods

#### Participants

Undergraduates from the University of Melbourne (129: 88 female; age range, 17–45, mean, 19.059) completed the tasks for course credit. Twelve participants were removed for errors in more than 20% of the tasks.

#### Materials, procedure, and design

The methods were the same as in Experiment 1a, but feedback was given regarding the correctness of the solution (this data is investigated in forthcoming papers). The affect-related questions were asked both before and after the solution feedback was given. In the current analysis, only the data from before accuracy feedback was used.

### Results and discussion

#### Differences in accuracy and Aha

As in the first two experiments, strong support for the effect of problem type on solution accuracy, *F*_(2, 224)_ = 7.964, *BF*_10_ = 47.61, η^2^ = 0.066, with significantly higher accuracy on non-insight problems compared to classic insight problems (mean difference = 11.02, *BF*_10_ = 36.184) and CRAs (mean difference = 8.94, *BF*_10_ = 15.040). There was no significant difference between insight problems and CRAs (mean difference = 0.02, *BF*_10_ = 0.133). This marks another change from both previous experiments: The accuracy across problem type is not consistent, but seems to follow a similar trend, with higher accuracy on non-insight problems, lower accuracy on insight problems.

As in the first two experiments, there was a significant difference in the reported insight in response to the problems *F*_(2, 205)_ = 5.370, *BF*_10_ = 4.389. As in Experiment 1a, this main effect was explained by the higher feelings of insight in response to classic insight questions, compared classic non-insight questions (mean difference = 6.13, *BF*_10_ = 4.679, see Figure 1B). Similarly replicating Experiment 1a, there was no significant difference either between reported insight between CRAs and insight problems (mean difference = 4.025, *BF*_10_ = 1.572). There was also no significant difference between non-insight problems and CRAs (mean difference = 2.105, *BF*_10_ = 0.199). This may again reflect the use of CRAs as hybrid problems. However, this inconsistency in differences in reported insight across problem types, even keeping the problems constant, flags potential problems in the use of non-insight problems as controls in insight problem studies, particularly without self-reported measures of insight.

## Correlational analyses

**Figure 4:**
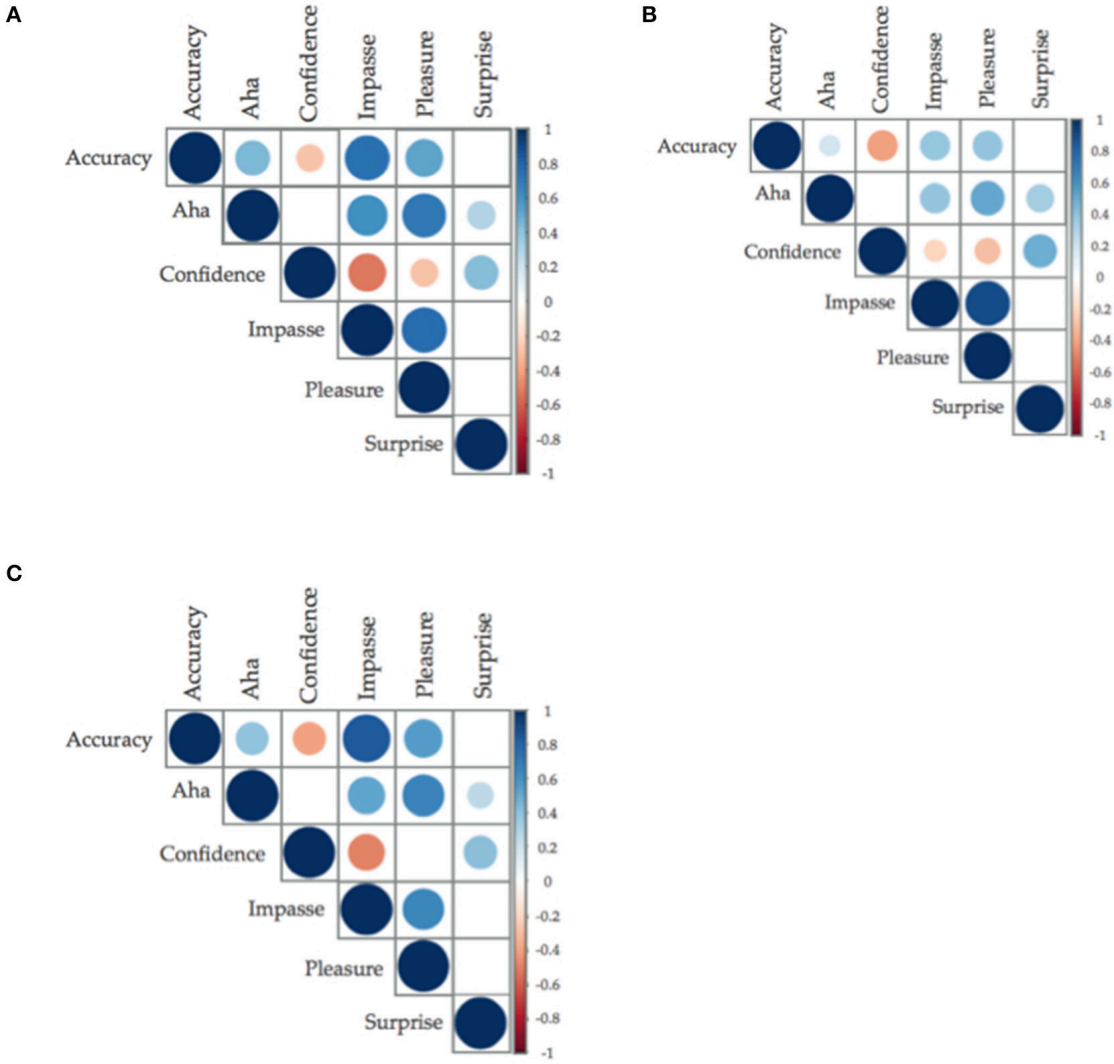
**(A–C)** Correlation plots between accuracy and insight and insight related affect (only data from before performance feedback was graphed). Size of the circle and saturation of color determine the strength of the correlation; the color determined the direction of the relationship, with positive being blue (**A**: Classic insight problems; **B**: classic non-insight problems; **C**: compound remote associates. Only relationships with less than *p* = 0.05 have been graphed).

As in Experiment 1a, feelings of Aha were significantly, and positively, correlated with accuracy across all problem types (insight problems: *r* = 0.495, *BF*_10_ > 150; CRAs: *r* = 0.39, *BF*_10_ > 150; non-insight problems: *r* = 0.19, *BF*_10_ = 40.007 (see Figure 4 for graphical representation, or Supplementary Materials for correlation matrices). This replication of the relationship between accuracy and Aha across all problem types emphasizes the requirement of self-report before use of non-insight problems as controls for insight problems.

The direction and strength of the relationships held for Experiment 2 within insight related affect. This consistency of a significant positive relationship with Surprise and Aha, compared to either a significant negative or non-significant relationship between Surprise and solution accuracy again indicates the importance of Surprise in the Aha experience.

## Multilevel analysis

We sought to determine how well accuracy could be predicted from the subjective feeling of insight along with the other measures recorded in our study: Impasse, Pleasure, Surprise, and Problem Type. Due to high collinearity between ratings of Confidence and Aha, we removed Confidence from the analysis. We used a multilevel logistic regression in order to account for different overall levels of accuracy for each subject (i.e., by including different subject level intercepts) and different levels of accuracy across problem types. We modeled the binary-valued accuracy as a logistic function of these variables. Data from native-English speaking participants from across Experiments 1, 1a, and 2, were combined for this analysis. (One question, the Trace non-insight problem was removed from Experiments 1a and 2 and is not analyzed here).

We compared a number of different multilevel models: The first model included the rated feelings of: Insight, Impasse, Pleasure and Surprise, as well as problem type and is given by the equation:

(1) $$\begin{array}{l} y_{ij}=\beta_{0}+\beta_{1}{\text{Insight}}_{ij}+\beta_{2}{\text{Impasse}}_{ij}+\beta_{3}{\text{Pleasure}}_{ij}+\\ \;\beta_{4}{\text{Surprise}}_{ij}+\beta_{5}{\text{Type}}_{ij}+(S_{i}+\varepsilon_{ij})\; \end{array}$$

where *y*_*ij*_ is the binary response accuracy indicating whether participant *i* make a correct (1) or incorrect (0) responses on item *j*. Each term in the model represents participant *i's* ratings on that trait for item *j*. Each model also includes a set of subject-specific random effects, *S*_*i*_, and an error term, ε_*ij*_. We additionally fit a second model, which allowed for an interaction between insight and problem type. This model is based on the grounds that classic insight problems and CRAs are proposed to elicit greater amounts of Aha than non-insight problems:

(2) $$\begin{array}{l} \begin{array}{lll} y_{ij}= & \;\beta_{0}+\beta_{1}{\text{Insight}}_{ij}+\beta_{2}{\text{Impasse}}_{ij}+\beta_{3}{\text{Pleasure}}_{ij}+\;\; & \end{array}\\ \;\beta_{4}{\text{Surprise}}_{ij}+\beta_{5}{\text{Type}}_{ij}+\beta_{6}{\text{Insight}}_{ij}\times {\text{Type}}_{ij}+(S_{i}+\varepsilon_{ij})\; \end{array}$$

For both models, we compared accuracy across insight, CRAs, and non-insight problems through the inclusion of a categorical Type variable. This allowed us to use non-insight questions as a baseline and extract separate weights for insight problems and CRAs. Additionally, for both models, we systematically tested alternative random effects by allowing intercept to vary by participant (Models 1 and 3), by allowing intercept and problem type to vary by subject (Model 2), and by allowing intercept and the insight by problem type interaction to vary by subject (Model 4). These comparisons allow for (a) different overall performance between participants, (b) different performance on each type of problem, and (c) different levels of insight on each problem type to be expressed between participants. We determined the preferred model using the Bayesian Information Criterion (BIC). The results are presented in Table 2.

Comparison of the BICs pointed to Model 4 which included the interaction between Insight and Problem Type both as a fixed and random effect as the preferred model. As in all models, as might be expected, insight had a positive effect on accuracy, but the experience of impasse decreased accuracy. Further contrasting effects were found for pleasure, which increased accuracy, and surprise, which decreased accuracy. Accuracy was poorer on insight problems than on CRAs or non-insight problems, respectively. We also found higher interactions between insight and CRAs than between insight and insight problems.

**Table 2:** **Estimated parameters (and standard errors) of multilevel modeling**.

**Parameters**	**Model 1**	**Model 2**	**Model 3**	**Model 4**
**FIXED EFFECTS**				
Intercept (β_0_)	0.06 (0.11)	0.06 (0.12)	0.02 (0.10)	0.10 (0.12)
Insight (β_1_)	**1.11 (0.06)**	**1.18 (0.06)**	**0.39 (0.10)**	**0.51 (0.14)**
Impasse (β_2_)	**−1.07 (0.05)**	**−1.16 (0.05)**	**−1.01 (0.05)**	**−1.08 (0.05)**
Pleasure (β_3_)	**1.05 (0.06)**	**1.11 (0.07)**	**1.02 (0.06)**	**1.11 (0.07)**
Surprise (β_4_)	**−0.14 (0.05)**	**−0.14 (0.05)**	**−0.15 (0.05)**	**−0.19 (0.05)**
Type (Insight) (β_5_)	**−0.78 (0.12)**	**−0.86 (0.14)**	**−0.66 (0.11)**	**−0.76 (0.13)**
Type (CRAs) (β_5_)	**−0.48 (0.10)**	**−0.50 (0.13)**	**−0.45 (0.09)**	**−0.44 (0.11)**
Insight × Type (Insight) (β_6_)			**0.48 (0.13)**	**0.51 (0.17)**
Insight × Type (CRAs) (β_6_)			**1.03 (0.11)**	**1.08 (0.17)**
**RANDOM EFFECTS VARIANCE**				
Intercept (S_0_)	1.14	1.62	1.12	1.23
Type (Insight) (s_1_)		1.00		
Type (CRAs) (s_1_)		1.76		
Insight × Type (No Insight) (s_2_)				1.03
Insight × Type (Insight) (s_2_)				0.88
Insight × Type (CRAs) (s_2_)				0.78
**EVALUATION**				
*df*	8	13	10	19
BIC	5839.0	5780.8	5771.3	5627.8

*All significant coefficients are shown in bold font. df, degrees of freedom; BIC, Bayesian Information Criterion*.

## Overall summary

The three studies demonstrate a difference in problem solving and ability to elicit insight in insight and non-insight problems; however, the patterns elicited through correlation analyses indicate a relationship between performance accuracy and insight across problem types, when selected for English proficiency. Particularly, the consistent occurrence of a significant positive relationship between reported Aha and non-insight problems is worthy of further investigation. The results of the multilevel regression indicate the importance of the problem type and components of insight (surprise, impasse, Aha) elicited in the accuracy of a problem solution. However, these results do not enable us to differentiate between a feeling of insight as co-occurring with a correct solution, and the process of arising at a solution through insight-problem-solving vs. analytic problem solving.

## General discussion

In this work, we investigated the relationship between insight and accuracy across three different problem types, comparing classic “insight,” “non-insight” and non-classic insight problems (CRAs). Insight related affect were predictive of correct solutions. We also reflected upon the comparison of insight and “non-insight” problems in the literature, finding that differences in reported insight between problem types make the distinctions in the literature seem valid; however, our secondary analysis revealed a consistent relationship between accuracy and insight ratings in non-insight problems, which emphasizes the issues in the comparison of insight and non-insight problems without self-report measures.

## Accuracy and insight

Salvi et al. (2012, 2016) that a solution accompanied by insight is more likely to be correct than a solution that is systematically and consciously deduced. That is, insightful problem solving is an *all or none* process, in which the problem solver arrives at solution through processing which is subthreshold to awareness, and therefore unconscious. The implication is that a solution that has been obtained through an insightful process is not consciously accessible until the process of problem solving has been completed and therefore solutions are more likely to be either correct or omitted. Salvi et al.'s (2016) data contrasts with Metcalfe's statement that feeling suddenly close to the solution often marks an incorrect solution (Metcalfe, 1986, p. 633). However, investigations of insight across experiments indicate that there was a greater proportion of problems correctly solved with insight than incorrectly solved with insight. The discrepancy may arise from the different self-report measures: Metcalfe used Feeling of Warmth (FOW) ratings, which were generated *before* the problem was solved and investigated pattern ratings; Salvi et al. (2016) used participant indications of within-experiment defined insight that were given *after* the problem was solved (i.e., participants agreed that the solution was sudden, surprising, and felt like a small Aha moment).

We asked participants to rate the strength of insight related affect, and were so able to investigate the relationships between accuracy and insight components on a continuous measure (as used in Danek et al., 2014a), compared to the more common dichotomous measures of insight^3^. Our results are congruent with Salvi et al. (2016): a feeling of *Aha* is associated with accuracy. Our data could be interpreted in a similar manner to Salvi et al. (2016); that is, that an Aha experience is elicited during an insightful problem solving *process*. However, our use of a rating scale rather than a binary response enables us to investigate the strength of an Aha experience, which varies with a moderate relationship with accuracy.

At this point we must raise the possibility of a distinction between a sudden insight as a *process* as opposed to an *affect*. Whether *post-hoc* self-reports of Aha reflect insightful processing is unclear. Our data indicate that the feeling of insight varies in strength and that the strength of an Aha is related to Surprise more than accuracy. Our results indicate that there are many components to problem solving that is accompanied by an Aha.

## Methodological implications

The use of classic insight problems as *pure, hybrid*, or non-insight problems arise primarily from the papers of Weisberg (1995) and Metcalfe and Wiebe (1987); however, there has been little investigation regarding the efficacy of these problem types to elicit insight. We found significant differences in the efficiency of problem types in eliciting Aha experiences in a direction that was as expected: *pure* insight problems elicited the greatest degree of Aha, then *hybrid* problems (CRAs), and finally non-insight problems elicited the lowest ratings of Aha. This may be a reflection of how *well-defined* a problem is (DeYoung et al., 2008). An ill-defined problem (i.e., an insight problem) may be more likely to result in a feeling of surprise in the solution, which is in turn related to an Aha experience.

A shift to using insight problems as *ill-defined* problems may help avoid a number of the issues in the literature. DeYoung et al. (2008) does not require subjective feedback regarding the feeling of insight as they use insight problems as stimuli that require restructuring, thereby acknowledging and utilizing the potential problems of these stimuli. Our experiments therefore compare *well-defined* to *ill-defined* problems. Well-defined problems contain sufficient information in the question to allow steady progress toward a solution (DeYoung et al., 2008), while ill-defined problems have insufficient information to allow for incremental progress and typically require restructuring in how the problem is approached (as in an insight problem). Thus, insight problems are used by DeYoung and colleagues as a subordinate set of ill-defined problems.

Nevertheless, the comparison of problems which have been defined as non-insight or insight problems or even well/ill-defined problems retain the problem of trying to verbally define the processes of interest, rather than relying on computational approaches to identify latent cognitive processes that underlie task performance (e.g., Hélie and Sun, 2010).

## Insight in problem solving

The majority of research conducted into the efficacy of insight-eliciting problems has been conducted on CRAs (see, e.g., Jung-Beeman et al., 2004; Kounios et al., 2006; Sandkühler and Bhattacharya, 2011; Wegbreit et al., 2012; Salvi et al., 2016). Our data is congruent the finding that CRAs are able to elicit feelings of insight but do not do so necessarily. Furthermore, our results indicate accuracy is a significant factor of the Aha response.

The current data support the use of successfully solved insight problems as measures of elicited insight, yet they also call for caution; the positive relationship between Aha and accuracy in insight problems is moderate, and by no means very strong. Our data also provides indications of insight problems solved without feelings of insight.

Despite their use as a control for insight problems in research (Murray and Byrne, 2001; Ash and Wiley, 2006; Fleck, 2008; Gilhooly et al., 2010; Wieth and Zacks, 2011; Wen et al., 2013; DeCaro et al., 2015), “non-insight” problems demonstrated a significant positive correlation when completed by students with high English proficiency. These results are comparable to those of Davidson (1995), who reported that 12–13% of non-insight problems indicated an insight pattern of FOW ratings. They are also comparable to Metcalfe (1986), who reported insight problems and anagrams showing both an insight and incremental pattern of analysis (Feeling of Knowing ratings), again indicating that problems can be solved both with feelings of insight, and by working through each step (Bowden, 1997; Weisberg, 2014).

The positive relationship between accuracy and Aha in insight and non-insight problems alike is congruent with the literature that indicates that problems can be solved with and without feelings of insight (Danek et al., 2016), and calls for the use of some form of self-report in all studies investigating insight affect and insight processes.

## Conclusion

The current study indicates that accuracy is often heralded by feelings of insight and insight-related affect (such as Confidence, and Pleasure). We have indicated that Surprise may be a significant indicator of Aha experiences, as it has a moderate to strong positive relationship to Aha experiences while only a weak relationship to solution accuracy.

Further, we have shown that both well-defined and ill-defined problems (or non-insight, and insight problems respectively) can be solved both with feelings of insight, and by consciously working through each step (Weisberg, 2014). Without participant driven feedback regarding feeling or occurrence of insight, the assumption that some problem types elicit insight, and are solved using particular processes (i.e., insightful or analytic) is highly problematic. While this data cannot tease apart whether feelings of insight in problem solving is indicative of a “special” and separate process, it does provide evidence for insight and insight-related quale in insight and non-insight problems.

## Author contributions

MW: Write up, running participants, analysis and design of experiment. DL: Data analysis (advice and running), write up. SC: Write up, experimental design.

### Conflict of interest statement

The authors declare that the research was conducted in the absence of any commercial or financial relationships that could be construed as a potential conflict of interest.

## Footnotes

^1^Scales 2:5 are drawn from the methodology of Danek et al. (2014b).

^2^Bayesian tests were computed using JASP (Love et al., 2015). The null hypothesis indicates that the effect size equals zero; the alternative hypothesis is that the effect size is not equal to 0 and is assigned a Cauchy prior (Rouder et al., 2009). Any Bayes factor less than 1 (reported BF < 1) indicates support for the null hypothesis (Kass and Raftery, 1995). Any BF > 150 indicates very strong support for the alternative hypothesis. 1 < BF < 3 provides weak evidence for the alternative hypothesis (“barely worth a mention,” Kass and Raftery, 1995, see also Jeffreys, 1961), 3 > BF > 20 is considered positive evidence for the alternative hypothesis, and 20 < BF < 150 is considered strong support for the alternative hypothesis.

^3^While in the examination of the frequency of responses on the response scale, we do see modes at the extreme values of 0 and 100; however, there is also substantial data that ranges between these values. For instance, 35% of the responses were in the inner quartile range and 70% of responses did not equal the extreme values. We therefore determined that dichotomising the continuous data would discard relevant information. Furthermore, the proportion of inner quartile range responses make it unclear where the cut-off should be between “no insight” and “insight.” Consequently, we determined that splitting the scale at 50 would be problematic since this would be grouping quite substantial feelings of aha into the no insight category.
