# Supplementary material for: Insight Is Not in the Problem: Investigating Insight in Problem Solving across Task Types
Source: Front Psychol. 2016 Sep 26;7:1424. doi: 10.3389/fpsyg.2016.01424 (PMC5035735; doi:10.3389/fpsyg.2016.01424)
Supplement: Supplementary file 1 [file Table1.DOCX]

**SUPPLEMENTARY MATERIALS**

**CORRELATION MATRICES CORRESPONDING TO THE CORRPLOTS**

**EXPERIMENT 1.** These correlations correspond to Figure 2. Insight problems = Figure 1a, Non-insight problems = Figure1b, Compound Remote Associates = Figure 1c

Table 1: Correlations between insight problems’ solving affect and accuracy (Figure 2a)

|  | Acc | Aha | Impasse | Confidence | Pleasure | Surprise |
| --- | --- | --- | --- | --- | --- | --- |
| Acc |  | .50*** | -.59*** | .73*** | .60*** | .11 |
| Aha |  |  | -.22* | .64*** | .73*** | .48*** |
| Impasse |  |  |  | -.68*** | -.42 | .26** |
| Confidence |  |  |  |  | .66*** | .08 |
| Pleasure |  |  |  |  |  | .26** |
| Surprise |  |  |  |  |  |  |
