# Supplementary material for: Insight Is Not in the Problem: Investigating Insight in Problem Solving across Task Types
Source: Front Psychol. 2016 Sep 26;7:1424. doi: 10.3389/fpsyg.2016.01424 (PMC5035735; doi:10.3389/fpsyg.2016.01424)
Supplement: Supplementary file 2 [file Table2.DOCX]

Table 2: Correlations between non-insight problem solving affect and accuracy (Figure 2b)

|  | Acc | Aha | Impasse | Confidence | Pleasure | Surprise |
| --- | --- | --- | --- | --- | --- | --- |
| Acc |  | .02 | -.58*** | .62*** | .27* | -.26* |
| Aha |  |  | .02 | .35** | .58*** | .40** |
| Impasse |  |  |  | -.64*** | -.25* | .30* |
| Confidence |  |  |  |  | .52 | -.12 |
| Pleasure |  |  |  |  |  | .23* |
| Surprise |  |  |  |  |  |  |
