# Supplementary material for: Insight Is Not in the Problem: Investigating Insight in Problem Solving across Task Types
Source: Front Psychol. 2016 Sep 26;7:1424. doi: 10.3389/fpsyg.2016.01424 (PMC5035735; doi:10.3389/fpsyg.2016.01424)
Supplement: Supplementary file 3 [file Table3.DOCX]

Table 3: Correlations between CRAs’ solving affect and accuracy (Figure 2c)

|  | Acc | Aha | Impasse | Confidence | Pleasure | Surprise |
| --- | --- | --- | --- | --- | --- | --- |
| Acc |  | .41*** | -.02 | .64*** | .46*** | -.09 |
| Aha |  |  | -.14 | .63*** | .70*** | .24* |
| Impasse |  |  |  | -.33** | -.06 | .18 |
| Confidence |  |  |  |  | .53*** | -.02 |
| Pleasure |  |  |  |  |  | .33** |
| Surprise |  |  |  |  |  |  |
