# Supplementary material for: Insight Is Not in the Problem: Investigating Insight in Problem Solving across Task Types
Source: Front Psychol. 2016 Sep 26;7:1424. doi: 10.3389/fpsyg.2016.01424 (PMC5035735; doi:10.3389/fpsyg.2016.01424)
Supplement: Supplementary file 4 [file Table4.DOCX]

**EXPERIMENT 1A.** These correlations correspond to Figure 3.

Table 4: Correlations between insight problems’ solving affect and accuracy (Figure 3a)

|  | Acc | Aha | Impasse | Confidence | Pleasure | Surprise |
| --- | --- | --- | --- | --- | --- | --- |
| Acc |  | .40** | -.29** | .75*** | .39** | -.26** |
| Aha |  |  | -.29** | .65*** | .73*** | .30** |
| Impasse |  |  |  | -.50*** | -.23* | .28* |
| Confidence |  |  |  |  | .60*** | -.15 |
| Pleasure |  |  |  |  |  | .11 |
| Surprise |  |  |  |  |  |  |
