# Supplementary material for: Insight Is Not in the Problem: Investigating Insight in Problem Solving across Task Types
Source: Front Psychol. 2016 Sep 26;7:1424. doi: 10.3389/fpsyg.2016.01424 (PMC5035735; doi:10.3389/fpsyg.2016.01424)
Supplement: Supplementary file 5 [file Table5.DOCX]

Table 5: Correlations between non-insight problems’ solving affect and accuracy (Figure 3b)

|  | Acc | Aha | Impasse | Confidence | Pleasure | Surprise |
| --- | --- | --- | --- | --- | --- | --- |
| Acc |  | .24* | -.40*** | .47** | .23 | -.16 |
| Aha |  |  | .05 | .41** | .56 | .49*** |
| Impasse |  |  |  | -.55*** | -.12 | .48*** |
| Confidence |  |  |  |  | .63*** | -.11 |
| Pleasure |  |  |  |  |  | .32* |
| Surprise |  |  |  |  |  |  |
