# Supplementary material for: Insight Is Not in the Problem: Investigating Insight in Problem Solving across Task Types
Source: Front Psychol. 2016 Sep 26;7:1424. doi: 10.3389/fpsyg.2016.01424 (PMC5035735; doi:10.3389/fpsyg.2016.01424)
Supplement: Supplementary file 6 [file Table6.DOCX]

Table 6: Correlations between CRA solving affect and accuracy (Figure 3c)

|  | Acc | Aha | Impasse | Confidence | Pleasure | Surprise |
| --- | --- | --- | --- | --- | --- | --- |
| Acc |  | .40** | -.34** | .72*** | .46*** | -.18 |
| Aha |  |  | -.25* | .60*** | .63*** | .15 |
| Impasse |  |  |  | -.63*** | -.17 | .41** |
| Confidence |  |  |  |  | .53*** | -.22 |
| Pleasure |  |  |  |  |  | .27* |
| Surprise |  |  |  |  |  |  |
