# Supplementary material for: Insight Is Not in the Problem: Investigating Insight in Problem Solving across Task Types
Source: Front Psychol. 2016 Sep 26;7:1424. doi: 10.3389/fpsyg.2016.01424 (PMC5035735; doi:10.3389/fpsyg.2016.01424)
Supplement: Supplementary file 7 [file Table7.DOCX]

**EXPERIMENT 2.** These tables correspond to Figure 4.

Table 7: Correlations between insight problems’ solving affect and accuracy (Figure 4a)

|  | Acc | Aha | Impasse | Confidence | Pleasure | Surprise |
| --- | --- | --- | --- | --- | --- | --- |
| Acc |  | .44*** | -.28** | .73*** | .52*** | -0.03 |
| Aha |  |  | -.17 | .60*** | .71*** | .29** |
| Impasse |  |  |  | -.52 | -.28 | .43*** |
| Confidence |  |  |  |  | .75*** | -.07 |
| Pleasure |  |  |  |  |  | .12 |
| Surprise |  |  |  |  |  |  |
