# Supplementary material for: Insight Is Not in the Problem: Investigating Insight in Problem Solving across Task Types
Source: Front Psychol. 2016 Sep 26;7:1424. doi: 10.3389/fpsyg.2016.01424 (PMC5035735; doi:10.3389/fpsyg.2016.01424)
Supplement: Supplementary file 8 [file Table8.DOCX]

Table 8: Correlations between non-insight problems’ solving affect and accuracy (Figure 4b)

|  | Acc | Aha | Impasse | Confidence | Pleasure | Surprise |
| --- | --- | --- | --- | --- | --- | --- |
| Acc |  | .19* | -.40*** | .38*** | .39*** | -.07 |
| Aha |  |  | -.10 | .39*** | .51*** | .33** |
| Impasse |  |  |  | -.21* | -.29* | .48*** |
| Confidence |  |  |  |  | .89*** | -.01 |
| Pleasure |  |  |  |  |  | -.01 |
| Surprise |  |  |  |  |  |  |
