# Supplementary material for: Insight Is Not in the Problem: Investigating Insight in Problem Solving across Task Types
Source: Front Psychol. 2016 Sep 26;7:1424. doi: 10.3389/fpsyg.2016.01424 (PMC5035735; doi:10.3389/fpsyg.2016.01424)
Supplement: Supplementary file 9 [file Table9.DOCX]

Table 9: Correlations between CRA solving affect and accuracy (Figure 4c)

|  | Acc | Aha | Impasse | Confidence | Pleasure | Surprise |
| --- | --- | --- | --- | --- | --- | --- |
| Acc |  | .39*** | -.40*** | .84*** | .55*** | -.13 |
| Aha |  |  | -.14 | .52*** | .65*** | .25** |
| Impasse |  |  |  | -.50*** | -.11 | .41*** |
| Confidence |  |  |  |  | .64*** | -.18 |
| Pleasure |  |  |  |  |  | .13 |
| Surprise |  |  |  |  |  |  |
