# Supplementary material for: Insight Is Not in the Problem: Investigating Insight in Problem Solving across Task Types
Source: Front Psychol. 2016 Sep 26;7:1424. doi: 10.3389/fpsyg.2016.01424 (PMC5035735; doi:10.3389/fpsyg.2016.01424)
Supplement: Supplementary file 10 [file Presentation1.PDF]

APPENDIX

Insight Problems

Tested in Karimi et al. (2007). The following three problems were classed as insight problems in the Karimi et al. (2007) paper.

Coin problem. A dealer of antique coins received an offer to buy a beautiful bronze coin by an unknown man. The coin had an emperor's head on one side and the date 544 B.C. stamped on the other side. The dealer examined the coin, but instead of buying it, he called the police to arrest the man. What made him realise that the coin was fake? *Solution*: BBC is a term used after Christ, not before

Egg problem. Using only a 7-minute and an 11-minute hourglass, how will you be able to time the boiling of an egg for exactly 15 minutes? *Solution*: (11-7)+11 = 15. So, start 11 and 7 simultaneously. When 7 has finished, start the egg boiling. When 11 has finished, flip it over for the remaining time.

Triangle of coins problem. Moving only 3 coins, make the triangle point downwards. *Solution*: move each of the corner coins to the middle of the following row.

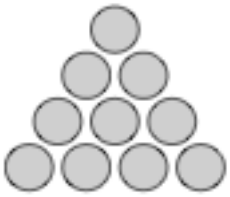

Drawn from Schooler et al. (1993).

Socks problem. If you have black socks and brown socks in your drawer, mixed in a ratio of 4 to 5, how many socks will you have to take out to make sure that you have a pair the same color? *Solution:* 3

Water lilies. Water lilies double in area every 24 hours. At the beginning of summer there is one water lily on the lake. It takes 60 days for the lake to become completely covered with water lilies. On which day is the lake half covered? *Solution*: 59

Non-insight Problems

Tested in Karimi et al. (2007). The following three problems were classed as non-insight problems in the Karimi et al. (2007) paper.

Card problem. Three cards from an ordinary deck are lying on a table, face down. The following information (for some peculiar reason) is known about those three cards (all the information below refers to the same three cards):

To the left of a queen there is a jack
To the left of a spade there is a diamond
To the right of a heart there is a king
To the right of a king there is a spade
Can you assign the proper suit to each picture card?

Solution: jack of hearts, king of diamonds, queen of spades.

Water jug problem. Given a source of unlimited water and 4 containers of different capacities – 99, 14, 25 and 11 – obtain exactly 86 L of water.
Solution: simplest: (25 x 3) + 11

Trace Problem. Without lifting pencil from paper, trace the figure provided below. A line cannot be traced more than once

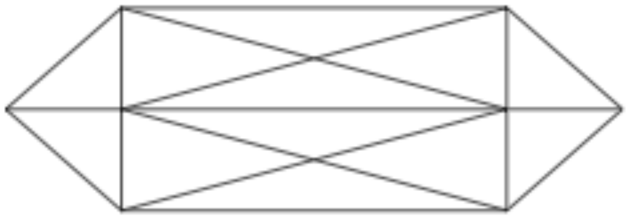

Drawn from Schooler et al. (1993).

Dinner. Dinner Mary won't eat fish or spinach, Sally won't eat fish or green beans, Steve won't eat shrimp or potatoes, Alice won't eat beef or tomatoes, and Jim won't eat fish or tomatoes. If you are willing to give such a bunch of fussy eaters a dinner party, which items from the following list can you serve: green beans, creamed codfish, roast beef, roast chicken, celery, and lettuce. *Solution:* roast chicken, celery, and lettuce.

Police. The police were convinced that either A, B, C, or D had committed a crime. Each of the suspects, in turn, made a statement, but only one of the four statements was true.

A said, “I didn't do it.”
B said, “A is lying.”
C said, “B is lying.”
D said, “B did it.”

Who is telling the truth? and
Who committed the crime?

Solution: B is telling the truth, and A committed the crime.
